# Exploring geographic distributions of high-risk water, sanitation, and hygiene practices and their association with child diarrhea in Uganda

**DOI:** 10.3402/gha.v9.32833

**Published:** 2016-10-26

**Authors:** Mitsuaki Hirai, Amira Roess, Cheng Huang, Jay Graham

**Affiliations:** 1 Department of Global Health, Milken Institute School of Public Health, George Washington University, Washington, DC, USA; 2 Department of Environmental and Occupational Health, Milken Institute School of Public Health, George Washington University, Washington, DC, USA

**Keywords:** WASH, water, sanitation, hygiene, Uganda

## Abstract

**Background:**

High-risk water, sanitation, and hygiene (WASH) practices are still prevalent in most low-income countries. Because of limited access to WASH, children may be put at an increased risk of diarrheal diseases.

**Objectives:**

This study aims to 1) develop a new measure of WASH-induced burden, the WASH Resource Index (WRI), and estimate its correlation with child diarrhea and an additive index of high-risk WASH practices; 2) explore the geographic distribution of high-risk WASH practices, child diarrhea, and summary indices at the cluster level; and 3) examine the association between the WRI and child diarrhea at the individual level.

**Design:**

A sample of 7,019 children from the Uganda Demographic and Health Survey 2011 were included in this study. Principal component analysis was used to develop a WRI, and households were classified as WASH poorest, poorer, middle, richer, and richest. A hot spot analysis was conducted to assess whether and how high-risk WASH practices and child diarrhea were geographically clustered. A potential association between the WRI and child diarrhea was examined through a nested regression analysis.

**Results:**

High-risk WASH practices were clustered at geographically distant regions from Kampala. The 2-week prevalence of child diarrhea, however, was concentrated in Eastern and East Central regions where high-risk WASH practices were not prevalent. At the individual level, none of the high-risk WASH practices were significantly associated with child diarrhea. Being in the highest WASH quintile was, however, significantly associated with 24.9% lower prevalence of child diarrhea compared to being in the lowest quintile (*p*<0.05).

**Conclusions:**

Only a weak association was found between the WRI and child diarrhea in this study. Future research should explore the potential utility of the WRI to examine WASH-induced burden.

## Introduction

Globally, almost 1,600 children under 5 years of age die from infections and malnutrition caused by diarrhea every day (1). In 2013, the under-5 mortality rate from diarrheal diseases was the highest in sub-Saharan Africa at 214 deaths per 100,000, compared to that of South Asia at 86 deaths per 100,000 (2). Limited access to water, sanitation, and hygiene (WASH) has been well recognized as a major risk factor for child diarrhea and mortality (3, 4). Yet, unsafe drinking water, inadequate sanitation, and limited hygiene are still attributable to most of the diarrhea-induced child mortality in Uganda, a low-income country in East Africa (2). This study examined Uganda as the focal country to highlight the importance of examining geographic disparities in WASH practices and guide public health actions at the subnational level.

Empirical research has elucidated the etiology and transmission mechanisms of diarrheal diseases in relation to WASH (5, 6). Diarrhea can be caused by bacteria, enteric viruses, protozoa, and helminths with varying levels of severity and symptoms (5); rotavirus is the most prevalent etiological agent globally (7). The fecal–oral transmission model, also known as the F-diagram, suggests that those infectious microorganisms in people's stools enter human bodies through the contact or consumption of contaminated fluids, foods, flies, fingers, and fields (6). Improving water supply, water quality, water storage, sanitation facilities, and handwashing behaviors can interrupt this transmission cycle (8) and reduce the risk of diarrhea by an estimated 17–48% (9, 10).

A large number of individuals, however, still engage in WASH practices that can increase diarrheal risks. Globally, 189 million people still rely on surface water as the main drinking water source, and 946 million people practice open defecation (11). Approximately 44% of the global population fetches water outside of the household, and water collection time has been found to be positively associated with an increased risk of child diarrhea (12). This is likely because when households are required to spend more than 30 minutes (min) to collect water, an adequate amount of water for basic needs (e.g. handwashing) may not be collected (13). The prevalence of handwashing with soap after using the toilet or after coming into contact with human excreta is also estimated to be 19% globally (14).

The global, regional, and national estimates of WASH coverage can highlight the magnitude of an issue, but they may mask large geographic disparities within a country (15). In Uganda, the proportion of the population who drinks surface water and practices open defecation is estimated to be 8 and 7%, respectively (11). These national averages, however, do not indicate where and how many high-risk WASH practices are geographically distributed in a community or a neighborhood. Consequently, the population bearing the highest level of WASH-induced burden may not be identified or supported with appropriate interventions. Few studies have examined the geographic distribution of WASH practices at smaller subnational geographic units to identify vulnerable populations and better target investments in the WASH sector (15).

Another gap in knowledge is the development and availability of measurement tools to identify the population bearing the highest WASH-induced burden. Although a few indices have been developed to examine WASH access and disparities (16–18), none of them specifically focus on high-risk WASH practices: drinking surface water without any treatment at point of use, open defecation, absence of a handwashing place for the household, and spending greater than 30 min for water collection. Moreover, the existing tools have been developed to assess WASH performance at the national level (16) and/or may require extensive resources (e.g. time and finance) for data collection (17). A new, affordable measurement tool therefore may be necessary to better identify populations with the least access to WASH resources.

This study addresses the stated knowledge gaps by 1) developing a new measure of WASH-induced burden, the WASH Resource Index (WRI); 2) exploring the geographic distribution of high-risk WASH practices, 2-week prevalence of child diarrhea, and summary indices at the cluster level; and 3) examining the association between the WRI and child diarrhea at the individual level. We hypothesized that high-risk WASH practices and the WRI are associated with child diarrhea after controlling for potential confounders.

## Methods

### Data source

This study analyzed data from the Uganda Demographic and Health Survey (DHS) 2011, a nationally representative household survey funded by the United States Agency for International Development (19). Since 1984, the DHS has been conducted in many low- and middle-income countries to obtain data on vital statistics and population health measures (20). To ensure the representativeness of the population in the survey, the DHS Program employs the stratified two-stage sampling method for sample selection (20). A country is typically stratified by the type of residence (urban/rural) and subnational regions to form strata. In the first stage of sampling, primary sampling units or clusters (e.g. a city block and village) are selected from each stratum based on probability proportional to size. In the second stage, households are systematically selected by referring to the household listing obtained in each cluster. Subsequently, trained interviewers visit selected households to invite adult women and men to voluntarily participate in the survey without any compensation; mothers report their children's demographic characteristics and health status on behalf of them. This sampling strategy and high response rates (i.e. households interviewed/households visited) contribute to a high level of generalizability of DHS study findings at the household and individual level (20). The Uganda DHS 2011 had a response rate of 95.3% (19). For this study, data on 7,208 children under the age of 5 were available in the child data set, and a list-wise deletion left 7,019 (97.4%) children for statistical analyses. The WRI was initially constructed with the household data set and merged with the child data set. Given that DHS data sets are publicly available, the Institutional Review Board (IRB) at the George Washington University determined that this study was exempted from the need of IRB approval or clearance.

### Variables

The dependent variable of this study was the 2-week prevalence of child diarrhea. Mothers reported if their children under 5 years of age had diarrhea during the 2 weeks prior to the survey. The independent variables of this study included 1) drinking surface water without any effective treatment, 2) open defecation, 3) absence of a place for handwashing in the household as a proxy for lack of handwashing with soap (21), 4) water collection time greater than 30 min, 5) an additive index of these four high-risk WASH practices, and 6) the WRI. Each of the high-risk WASH practices was a binary variable (1=Yes, 0=No). If households mentioned river, stream, pond, lake, or dam as the main drinking water source and did not report making the water safer with effective methods (e.g. boiling, using chlorine, and water filter) in the survey, they were regarded as drinking surface water without any treatment. Open defecation was measured with the question concerning the type of sanitation facilities that household members usually use, and households reporting no facility, bush, or field were counted as practicing this high-risk behavior (11). The handwashing variable was the only variable measured by direct observation. If the interviewer did not observe a specific location for handwashing in the home, yard, or plot during the data collection, households were considered as having no handwashing place. Self-reported water collection time was categorized into two timeframes (1=greater than 30 min, 0=30 min or less). All of these high-risk WASH practices were summed to create an additive index.

The WRI was constructed with the main source of drinking water, types of household sanitation facilities, practice of sharing sanitation facilities, handwashing materials in the household, and water collection time. All of the answer options in these variables except for ‘don't know’ or missing were included as indicator variables. A list of variables included for the construction of the WRI is summarized in Table 1. Principal component analysis (PCA) was used to summarize these WASH resource indicators into one summary measure – the methods used are the same as those for developing a wealth index in the DHS (22). Based on the WRI scores, the WASH quintiles were constructed to classify households with the categories of WASH poorest, poorer, middle, richer, and richest.

**Table 1:** A list of indicator variables used for the construction of the WASH Resource Index

DHS questions	Indicator variables
What is the main source of drinking water for members of your household?	1. Piped into dwelling 2. Piped to yard/plot 3. Public tap/standpipe 4. Open well/spring in yard/plot 5. Open public well/spring 6. Protected well/spring in yard/plot 7. Protected public well/spring 8. Borehole in yard/plot 9. Public borehole 10. River/stream/pond/lake/dam 11. Rain water 12. Tanker Truck 13. Vendor 14. Bottled water 15. Other
How long does it take to go there, get water, and come back?	16. Over 30 min
What kind of toilet facility do members of your household usually use?	17. Flush or pour flush toilet 18. VIP latrine 19. Covered pit latrine without slab 20. Covered pit latrine with slab 21. Uncovered pit latrine without slab 22. Uncovered pit latrine with slab 23. Composting toilet 24. No facility/bush/field 25. Ecosan 26. Other
Do you share this toilet facility with other households?	27. Shared
Please show me where members of your household most often wash their hands.	28. Handwashing place observed 29. Not in dwelling/yard/plot 30. No permission to see 31. Not observed for other reason
Observe presence of water at the place for handwashing.	32. Presence of water observed
Observe presence of soap, detergent, or other cleansing agent.	33. Presence of soap or detergent observed 34. Presence of ash, mud, or sand observed

DHS, Demographic and Health Survey.

Other independent variables included urban or rural residence, maternal education, and household wealth levels as proxy measures of sociodemographic characteristics. The wealth index in the DHS was constructed with water and sanitation variables, which can introduce a bias to the estimated association between the WRI and child diarrhea. Although empirical evidence suggests that wealth indices with or without water supply and sanitation variables are highly correlated and produce mostly concordant results in quintile assignments (23), the present study developed a wealth index without water and sanitation variables. The index included 14 types of household assets other than WASH-related assets to ensure the theoretical distinction. Control variables for the analysis were child age, child sex, sub-country regions, and month of the interview as a proxy for season.

### Statistical and spatial analysis

The prevalence of the aforementioned high-risk WASH practices, indices, and child diarrhea was estimated at the individual level and the cluster level. Each cluster consists of 2–38 children and represents the average value of these children. By using all of the clusters as data points, descriptive analyses were displayed on maps. This study also examined the correlation of an additive index of high-risk WASH practices and the WRI with child diarrhea to assess the performance of WRI as a proxy measure of WASH-related burden at the cluster level.

This study also conducted a hot spot analysis of high-risk WASH practices and child diarrhea. A hot spot analysis estimated the Getis-Ord Gi* statistic and *z*-scores to assess whether high-risk WASH practices and child diarrhea are geographically clustered (24). More specifically, this analytical approach compared the local mean (e.g. average prevalence of open defecation for a few clusters including a cluster of interest) with the global mean (e.g. average prevalence of open defecation for all clusters). If the local mean was statistically higher or lower than the global mean, a given cluster was classified as a hot spot or a cold spot. The boundary of hot and cold spots was determined by the fixed distance method by which clusters within a critical distance form a bundle, and the influence from other clusters outside of this bundle will be zero (25). A fixed distance was set for an estimated local mean to include at least two clusters.

Bivariate and multivariate associations between independent variables and child diarrhea were examined by the generalized linear model (GLM) with Poisson family and log link. For the multivariate analysis, a nested regression model was employed with the WRI as the main independent variable. The zero-order model included the WRI and control variables. The second model added the type of residence (urban/rural) to the zero-order model. The third model added maternal education to the zero-order model. The fourth model added wealth quintiles to the zero-order model. The fifth model added all of the independent variables. By constructing multivariate models as a nested structure, the moderation effect of the type of residence, maternal education, and wealth levels on the association between the WRI and child diarrhea can be examined. Statistical analyses were adjusted for the complex survey design of the DHS by applying sampling weights, and fit statistics were estimated with adjusted Wald tests to identify the most parsimonious model.

## Results

Descriptive statistics of sociodemographic characteristics and high-risk WASH practices are presented in Table 2. Approximately 6 and 11% of children lived in households that reported drinking untreated surface water and engaging in open defecation practices, respectively. The majority of children did not have a location for handwashing available, and 41.3% lived in the households reporting to spend more than 30 min for water collection. Almost one in four children (24.1%) was reported to have had diarrhea during the 2 weeks prior to the survey.

**Table 2:** Sociodemographic and WASH characteristics

Variables	% of Children (*n*=7,019)
High-risk WASH practices	
Drinking unsafe water	6.13
Open defecation	11.13
Absence of handwashing place	56.95
Water collection time ≥ 30 min	41.30
Two-week prevalence of diarrhea	24.08
WASH Resource Index	
WASH poorest	6.11
WASH poorer	38.60
WASH middle	26.18
WASH richer	19.61
WASH richest	9.50
Wealth quintiles	
Poorest	23.13
Poorer	23.17
Middle	20.39
Richer	17.93
Richest	15.38
Region	
Kampala	5.86
Central 1	9.69
Central 2	10.42
East Central	11.25
Eastern	17.02
North	9.18
Karamoja	3.82
West-Nile	5.90
Western	14.64
Southwest	12.21
Type of residence	
Urban	14.06
Rural	85.94
Maternal education	
No education	14.42
At least some primary	63.61
At least some secondary	18.36
Higher	3.61
Age of children	
Less than 12 months	22.09
12–23 months	19.99
24–35 months	20.14
36–47 months	19.32
48–59 months	18.47
Sex of children	
Male	49.88
Female	50.12

WASH, water, sanitation, and hygiene.

About 6.1% of children belonged to the WASH poorest group (according to the WRI), potentially bearing the highest burden from inadequate access to WASH resources. The largest proportion of children was in the WASH poorer group at 38.6%. More than 85% of children lived in rural areas, and 14.4% of mothers did not have any formal education. Male and female children were almost equally represented in this study.

### Spatial analysis

**Fig. 1:**
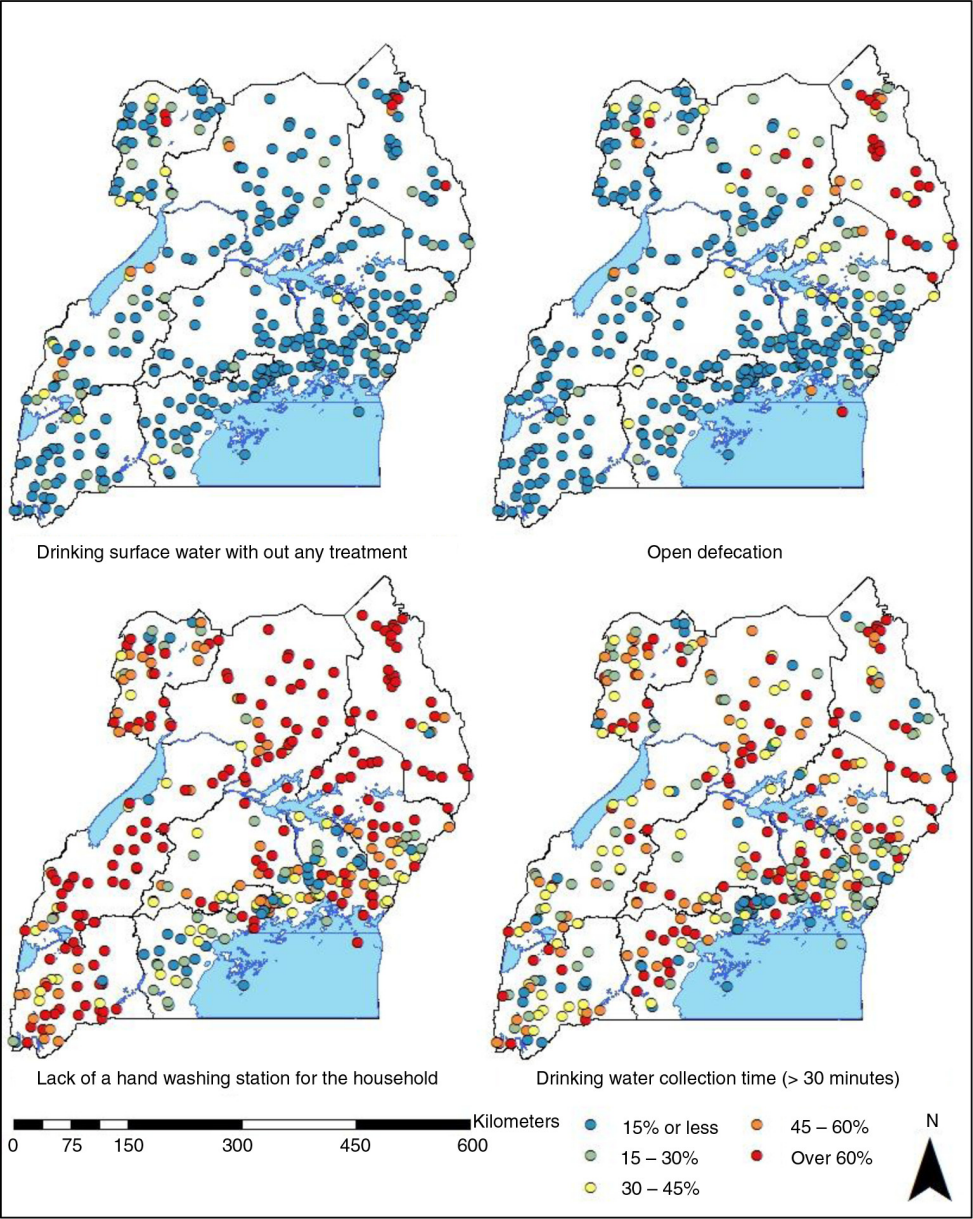
Geographic distributions of high-risk WASH practices at the cluster level in Uganda.

The geographic distribution of drinking untreated surface water, open defecation, lack of a handwashing place, and water collection time over 30 min at the cluster level is presented in Fig. 1. In most of the clusters, the proportion of children who were exposed to untreated surface water was below 15%, but in multiple clusters in Karamoja and West Nile regions this went over 60%. Regions that were further away from Kampala had more clusters with a higher prevalence of this high-risk practice than other regions. Many clusters in Karamoja region had high open defecation prevalence of over 60%, suggesting that a large proportion of children did not have access to sanitation facilities. A few clusters with the prevalence of over 60% are also located in North and West Nile regions and an island on Lake Victoria. In the South West region, all of the clusters except for one were estimated to have an open defecation prevalence lower than 15%. Compared to other high-risk WASH practices, the absence of a handwashing place for the household was prevalent across many sub-country regions. In many clusters, over 80% of children did not have immediate access to a handwashing place. In the western part of Central 1 region, however, a series of clusters had a prevalence below 20%. The map displays that a large proportion of children lived in the households spending over 30 min to obtain their drinking water without a clear geographic concentration of the practice.

**Fig. 2:**
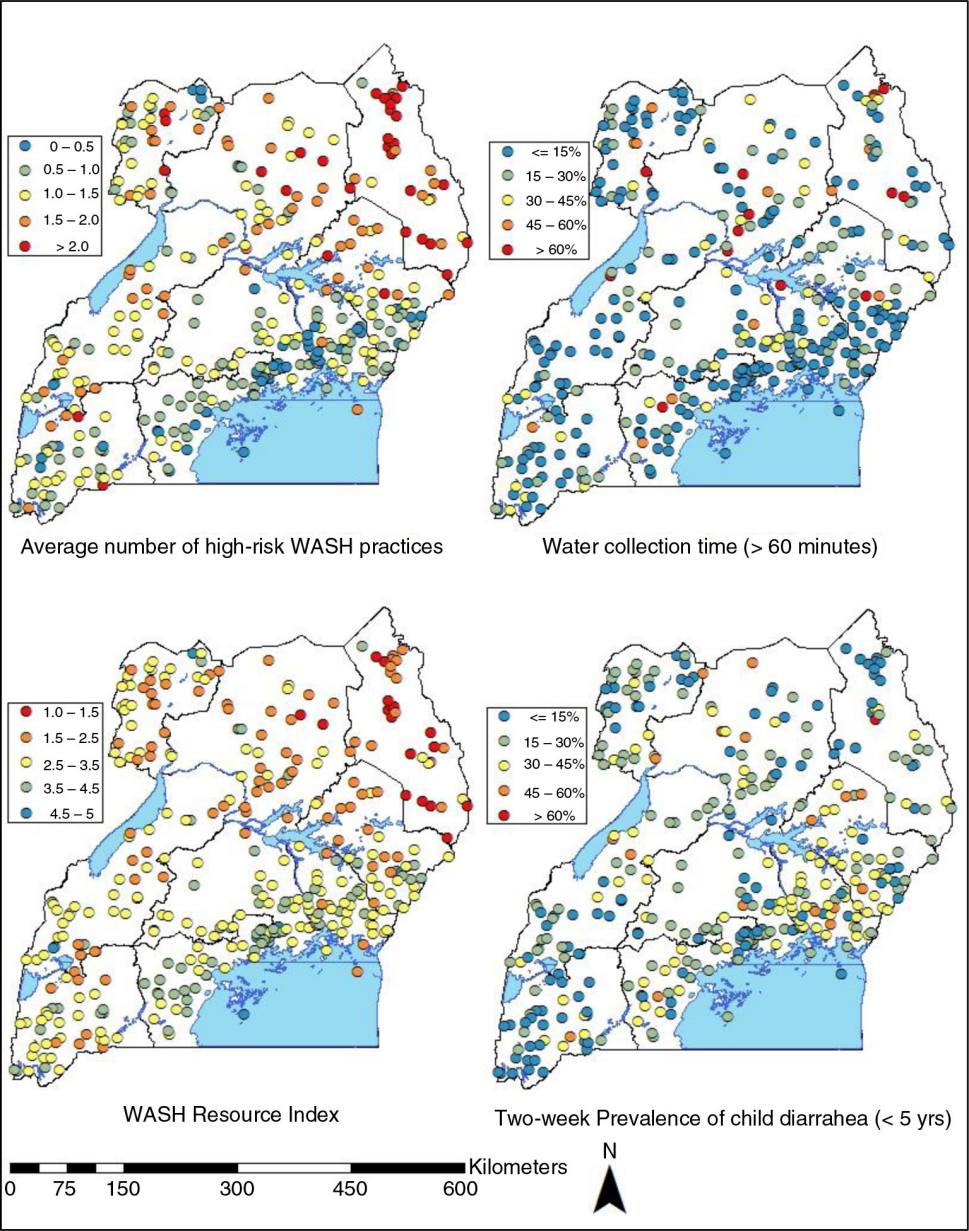
Geographic distributions of the average number of high-risk WASH practices, water collection over 60 min, WASH Resource quintiles, and 2-week prevalence of child diarrhea at the cluster level in Uganda.

Figure 2 displays the average number of high-risk WASH practices, self-reported water collection time of over 60 min, the average of WRI quintiles, and 2-week prevalence of child diarrhea at the cluster level. Many clusters in Karamoja region were exposed to at least two high-risk WASH practices on average. The map also shows that compared to Central regions (Central 1 and Central 2), clusters in geographically distant regions, such as West Nile, North, and Western, had a higher number of high-risk WASH practices. The proportion of children living in the households that spend more than one hour to collect their drinking water was lower than 15% in the majority of clusters. At some clusters, however, over 60% of children were estimated to engage in this practice without a clear geographic concentration. A series of clusters with the average child diarrhea prevalence of 30–45% were present in Eastern and East Central regions, but every region included multiple clusters with this level of diarrhea prevalence. Karamoja included many clusters with a high prevalence of drinking untreated surface water, open defecation, lack of handwashing place, and water collection time of over 30 min, but a fewer number of clusters had a high level of child diarrhea in the region.

**Fig. 3:**
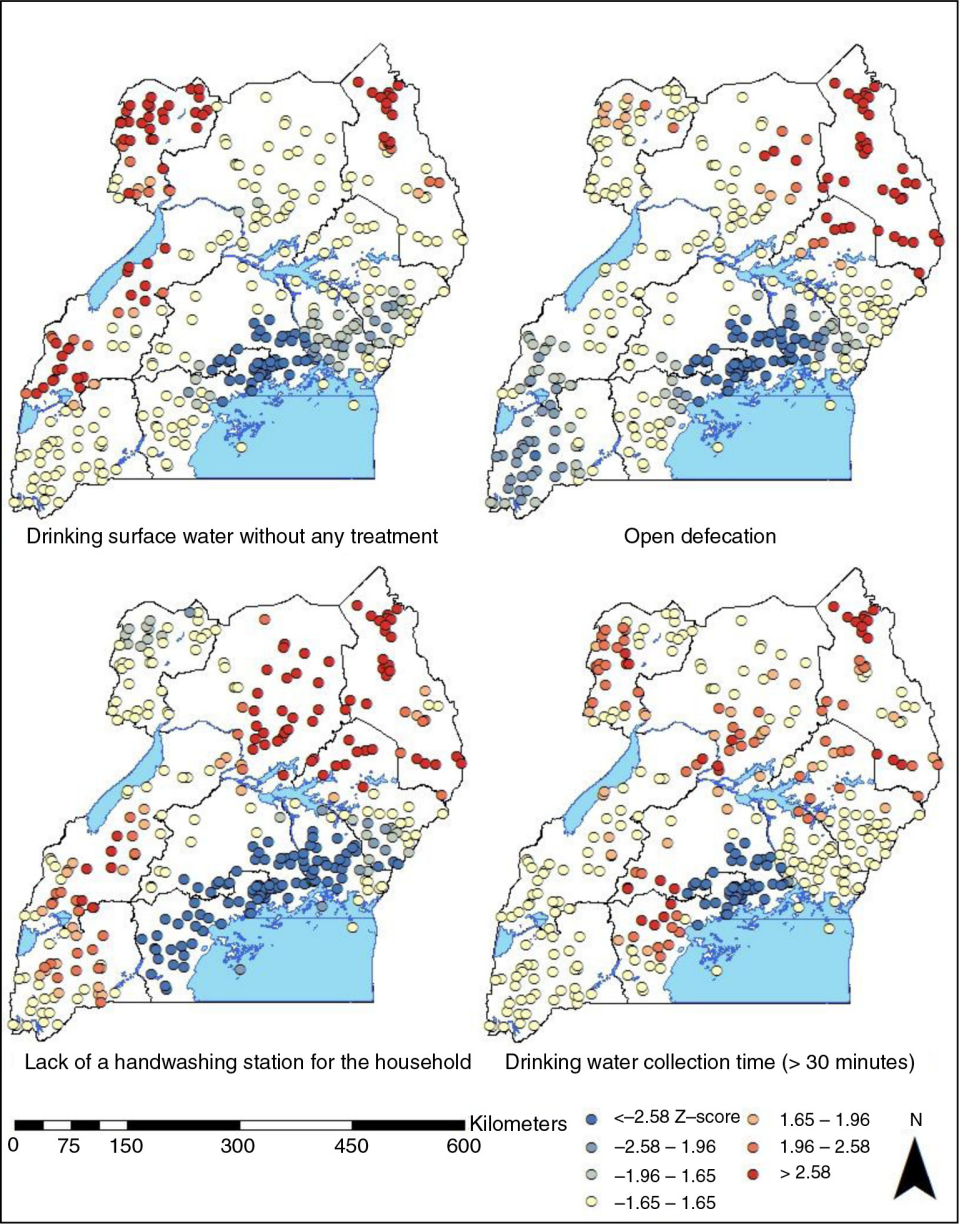
A hot spot analysis of high-risk WASH practices at the cluster level in Uganda.

The results of a hotspot spot analysis on high-risk WASH practices are presented in Fig. 3. Kampala was consistently classified as a cold spot (*z*-score<−1.96) for all of the high-risk WASH practices in this study. Hot spots of drinking surface water without effective treatment (*z*-score>1.96) were located in West Nile, Western, and Karamoja regions. All of the clusters in Karamoja region and a few clusters from West Nile, North, and Eastern regions represent hot spots of open defecation. Western, North, Eastern, and Karamoja regions also included hot spots of lack of handwashing place in the household. Hot spots of time-consuming water collection labor were located in most of the sub-country regions except for Southwest.

**Fig. 4:**
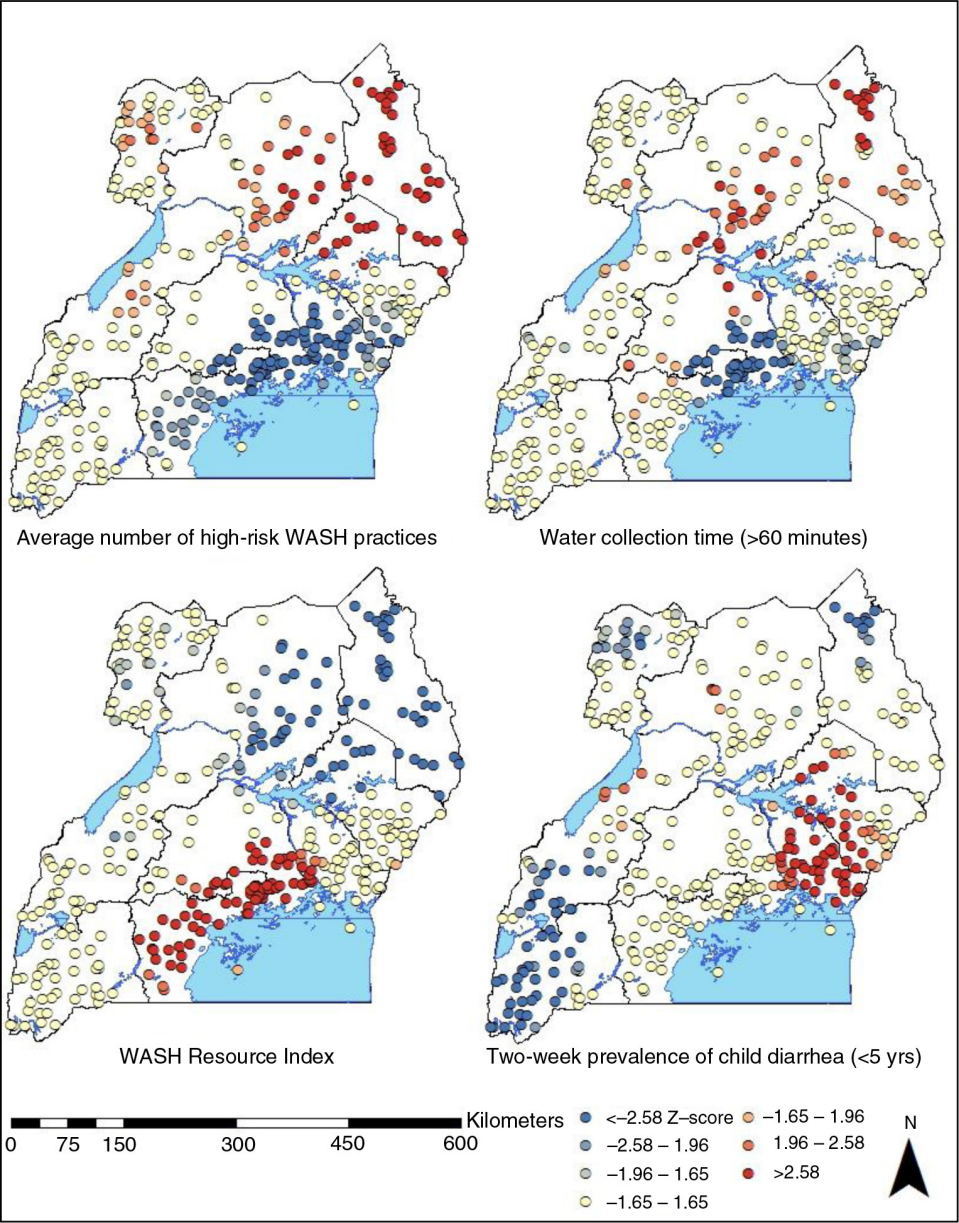
A hot spot analysis of the average number of high-risk WASH practices, water collection over 60 min, WASH Resource quintiles, and 2-week prevalence of child diarrhea at the cluster level in Uganda.

Figure 4 presents the hot spots and cold spots of the average number of high-risk WASH practices, water collection over 60 min, WRI quintiles, and 2-week prevalence of child diarrhea at the cluster level. The analysis suggests that communities and villages in Karamoja, North, West Nile, and Eastern regions engage in a significantly higher average number of high-risk WASH practices than the global mean or the national average of 1.19 (*p*<0.05). Hot spots of water collection time of greater than 60 min were mostly concentrated in the southern part of North region and the northern part of Karamoja region. Many clusters in Kampala and Central regions were highlighted as the hot spots of the average WRI quintiles, suggesting that children with higher or richer quintiles were clustered in these geographic areas. Hot spots of child diarrhea are clustered in Eastern and East Central regions, and cold spots were located in Karamoja, West Nile, Western, and Southwest regions. The results suggest that hot spots of high-risk WASH practices and child diarrhea do not overlap with each other. A few clusters located in the northern part of Karamoja region were consistently classified as hot spots of high-risk WASH practices, but they were cold spots of child diarrhea.

### Assessment of WRI

**Fig. 5:**
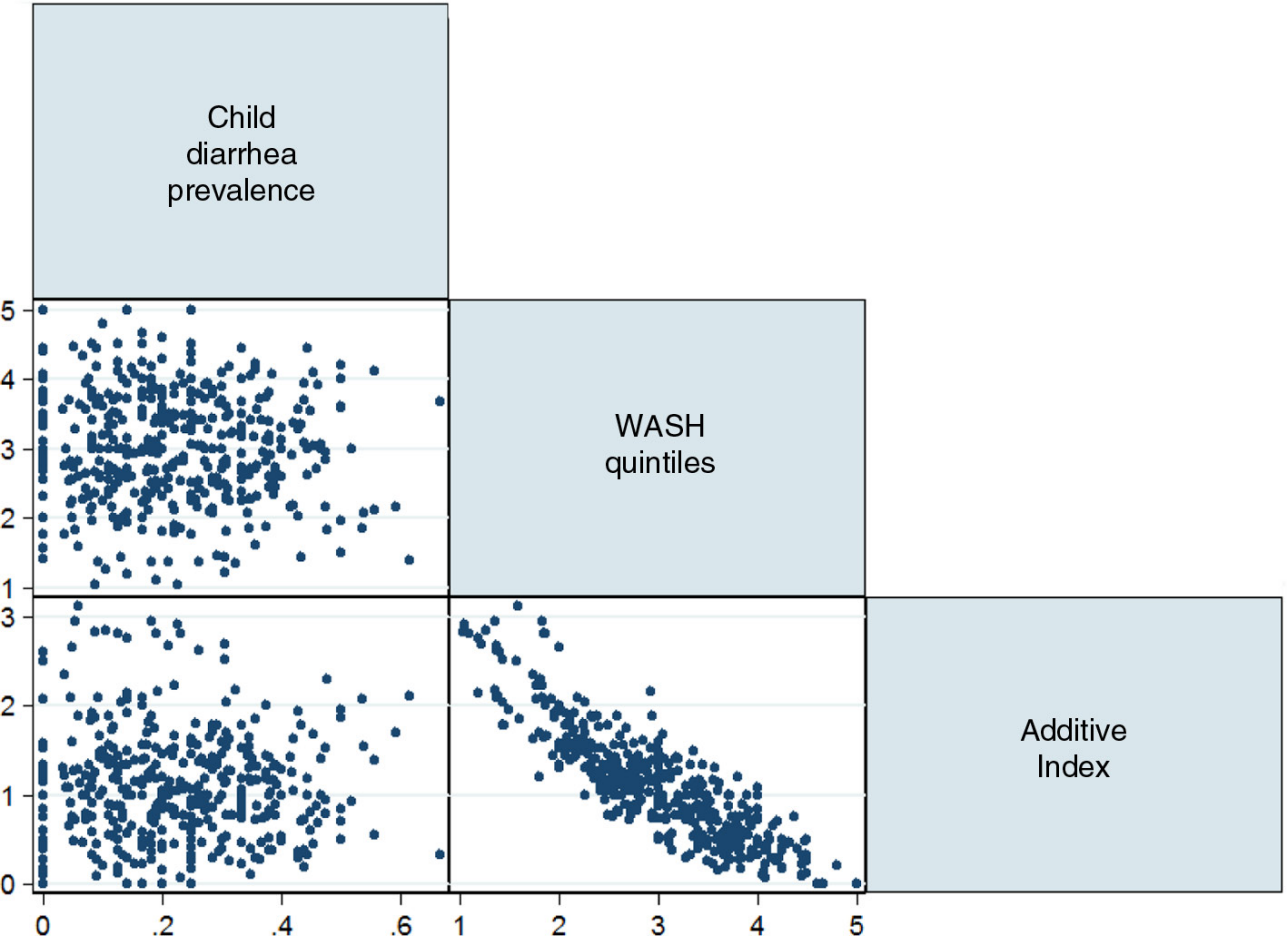
A scatter plot matrix of 2-week prevalence of child diarrhea, the average number of high-risk WASH practices, and the average WRI quintiles at the cluster level.

A scatter plot matrix on the 2-week prevalence of child diarrhea, the average number of high-risk WASH practices, and the mean WASH quintiles from the calculated WRI at the cluster level is presented in Fig. 5. The additive index of high-risk WASH practices and the WASH quintiles were both negatively associated with child diarrhea (*p*<0.001) without a clear linear relationship.

Based on PCA, the interpretability of the principal component in WRI was limited. A high negative correlation (*r*=−0.86) with the average number of high-risk WASH practices, however, suggests that children in higher quintiles of WRI (e.g. WASH Richest) may be exposed to a fewer number of high-risk WASH practices. Thus, the WRI quintiles may be a useful summary proxy measure of WASH-related burden from limited access to WASH resources at least in this context.

### Bivariate and multivariate analysis

In accordance with the findings from the spatial analysis, this study did not find a statistically significant bivariate association between high-risk WASH practices and child diarrhea (Table 3). Compared to the lowest quintile in the WRI, the highest quintile was associated with 24.9% lower prevalence (*p*<0.05). The WRI quintiles, however, were not significantly associated with child diarrhea.

**Table 3:** Unadjusted prevalence ratios (PR) of child diarrhea by high-risk WASH practices and WRI quintiles

Variables	PR	Std. err.	*t*	95% CI		df	*F*
High-risk WASH practices							
Drinking untreated surface water	0.879	0.998	−1.13	0.704	1.099	(1, 385)	1.28
Open defecation	1.151	0.099	1.64	0.972	1.363	(1, 385)	2.68
No handwashing place	0.892	0.050	−2.02	0.798	0.997	(1, 385)	4.09
Water collection time ≥30 min	1.096	0.061	1.64	0.982	1.223	(1, 385)	2.69
Water Resource Index quintiles						(4, 382)	1.30
WASH poorer	0.858	0.088	−1.49	0.701	1.051		
WASH middle	0.822	0.096	−1.68	0.654	1.034		
WASH richer	0.881	0.105	−1.06	0.698	1.113		
WASH richest	0.751*	0.103	−2.09	0.573	0.982		

PR, prevalence ratios; WASH, water, sanitation, and hygiene.

* *p*<0.05. WASH, water, sanitation, and hygiene.

The results of a multivariate analysis at the individual level are presented in Table 4. In Model 1, children in WASH middle, richer, and richest quintiles were associated with significantly lower diarrhea prevalence than that of WASH poorest quintile by 20.0, 21.0, and 25.7%, respectively (*p*<0.05). Adding rural residence in Model 2, maternal education in Model 3, and wealth quintiles in Model 4 completely mediated the relationship between WRI quintiles and child diarrhea. The first three models suggested that moving up the WRI quintile is associated with a progressively lower prevalence of child diarrhea prevalence, but this trend was not detected in Model 4 and Model 5.

**Table 4:** Adjusted prevalence ratios of child diarrhea by GLM with Poisson family and log link function

	Model 1	Model 2	Model 3	Model 4	Model 5
WASH Resource Index (Ref=Poorest)					
WASH poorer	0.853	0.856	0.871	0.890	0.897
WASH middle	0.800*	0.828	0.830	0.881	0.880
WASH richer	0.790*	0.806	0.824	0.858	0.866
WASH richest	0.743*	0.779	0.820	0.863	0.888
Sub-country regions (Ref=Kampala)					
Central 1	0.769	0.653*	0.686*	0.625*	0.629**
Central 2	0.81	0.705*	0.723*	0.657**	0.657**
East Central	1.161	0.998	1.028	0.894	0.903
Eastern	0.995	0.851	0.877	0.705*	0.717*
North	0.861	0.742	0.745	0.590**	0.596**
Karamoja	0.703	0.608*	0.618*	0.473***	0.492**
West-Nile	0.640**	0.550***	0.552***	0.434***	0.439***
Western	0.691*	0.598**	0.610**	0.530***	0.536***
Southwest	0.479***	0.408***	0.417***	0.361***	0.365***
Child age (Ref=Less than 12 months)					
12–23 months	1.200**	1.199**	1.202**	1.214**	1.215**
24–35 months	0.710***	0.707***	0.707***	0.717***	0.715***
36–47 months	0.486***	0.487***	0.489***	0.490***	0.492***
48–59 months	0.343***	0.343***	0.340***	0.345***	0.344***
Female (Ref=Male)	0.921	0.922	0.918	0.922	0.921
Rural residence (Ref=Urban)		1.200			0.955
Maternal education (Ref=No educ)					
Primary			1.020		1.054
Secondary			0.798*		0.880
Higher			0.503***		0.589*
Wealth quintiles (Ref=Poorest)					
Poorer				0.874	0.885
Middle				0.756**	0.766**
Richer				0.783**	0.820*
Richest				0.560***	0.630**
Constant	0.663*	0.648*	0.754	0.972	0.963
Model fit					
F-statistic	17.93	17.81	18.85	18.03	17.45
df	(26, 360)	(27, 359)	(29, 357)	(30, 356)	(34, 352)

All models controlled for the month of interview, number of household members, and rooms used for sleeping.

* *p*<0.05

** *p*<0.01

*** *p*<0.001. WASH, water, sanitation, and hygiene.

Compared to Kampala, children in West Nile, Western, and Southwest regions were significantly associated with lower diarrhea prevalence in all of the models. Child sex and rural residence were not associated with child diarrhea in multivariate models, but age was identified as a significant explanatory factor. One-year-old children were associated with significantly higher prevalence of child diarrhea than that of infants (*p*<0.01), but older children (>23 months) were associated with lower diarrhea prevalence (*p*<0.001). Maternal education and household wealth levels were also found to be significantly associated with lower child diarrhea. Based on the fit statistics, Model 3 was the most parsimonious model to examine child diarrhea prevalence in this study.

## Discussions

This study conducted spatial and statistical analyses to assess how high-risk WASH practices – drinking surface water without any effective treatment, open defecation, lack of handwashing place, and water collection time of 30 min or more – geographically distributed in Uganda and how these WASH factors were associated with 2-week prevalence of child diarrhea at the individual and cluster level. The WRI was developed as a new measurement tool to summarize household WASH resources and represent a level of burden from limited access to safe drinking water, adequate sanitation, and good hygiene.

### Opportunities with the WRI

Although this study employed PCA to develop the WRI, an additive index and the WRI produced mostly concordant results in their geographic distributions and correlations with child diarrhea. This finding suggests that the most vulnerable population with WASH-induced burden may be adequately identified with the additive index, which can be developed without a complex procedure. Bivariate and multivariate analyses also revealed that the estimated prevalence ratio of child diarrhea by WASH quintiles differs only to a small extent, and the relationship between the WRI and child diarrhea was not linear. Accordingly, the WRI may not be the most efficient tool to examine the 2-week prevalence of child diarrhea in this context.

Despite the limited association found between the WRI and child diarrhea, ample opportunities exist to examine the potential utility of the WRI in future investigations. First, the WRI can be used to assess WASH-related child health outcomes, such as stunting, respiratory infections, and soil-transmitted helminths. By estimating the prevalence of such health issues among the WASH poorest, targeted interventions can be delivered to the population with the highest public health need. Second, the WRI can be reproduced and used to identify the WASH poorest in other low- and middle-income countries. The DHS has employed a standardized questionnaire to collect the same variables across participating countries (26). By applying the same statistical and mapping methods from this study, other DHS countries may be able to identify the geographic area and the population potentially bearing the high level of WASH-induced burden.

### Implications of spatial analysis

The spatial analyses of high-risk WASH behaviors suggested that lack of a designated handwashing place for the household was particularly prevalent across the country. Handwashing behaviors can be shaped by many determinants including sociodemographic, psychosocial, and structural factors (27). Accordingly, having a handwashing place with water and soap may not immediately translate to handwashing practices. Nonetheless, it remains vital to enhance access to handwashing resources for the household as a physical cue to the behavior (28). An in-depth discussion on the behavioral determinants of handwashing with soap is beyond the scope of this paper, but the importance of access to infrastructure or technology to elicit handwashing behaviors has been well-recognized in literature (28, 29).

Another high-risk WASH practice, spending greater than 30 min for water collection, was also found to affect a large proportion of children throughout the country. Although 70% of the Ugandan population is estimated to have access to improved sources of drinking water (19), the reported water collection time remained high in this study. This finding suggests that people still bear a great deal of physical burden and opportunity costs of carrying water from the source to the household every day. Further efforts to promote access to drinking water on premise are essential as time-consuming water collection labor is pertinent to child health, school education, and maternal health.

The spatial analysis also suggested that high-risk WASH practices were clustered in Karamoja region while displaying a relatively low prevalence of child diarrhea. Empirical evidence suggests that WASH behaviors can be shaped by multiple levels of influence, such as sociocultural contexts, policies and regulations, physical environment, and personal values (30). Thus, some of these behavioral determinants could temporarily compel respondents in Karamoja region to engage in high-risk WASH practices. Poverty and maternal education could also partially account for this finding. A further analysis of the data found that 78.8% of children in Karamoja region were from households in the poorest wealth quintile, and 64.0% of mothers had not received any formal education. Accordingly, people's ability to purchase and maintain WASH-related resources was probably limited in this region. Limited education to learn about diarrheal disease at school could have also rendered some mothers to underreport children with diarrhea. Previous research suggested that child diarrhea was reported at increased rates in DHS by mothers with higher levels of education, which may also explain our findings (31).

A high prevalence of child diarrhea was found in the Eastern and Eastern Central regions despite a low concentration of high-risk WASH practices. A previous study in Uganda, which analyzed DHS data from 2000 to 2001, also suggested that these geographic areas had a higher prevalence of child diarrhea than other regions (32). The same study mentioned political instability as a potentially influential factor of this finding (32). The exact reasons for a high prevalence of child diarrhea in the Eastern region, however, remain unclear. Additional research is necessary to identify the most influential determinants of child diarrhea in this region and reduce the burden of diarrheal diseases.

### Limitations

There were a number of limitations of this study. First, only the presence of a handwashing place was observed; all of the other variables for this study were self-reported, so these may suffer from recall and reporting biases. Self-reported child diarrhea can be measured at different recall periods including 24 hours, 2 days, 7 days, and 2 weeks (33). Empirical evidence suggests that the 1-week recall period can provide reasonable estimates of diarrhea prevalence without requiring a significant increase in sample size or producing reporting bias (33). The major household surveys, including DHS and the Multiple Indicator Cluster Survey, however, still estimate 2-week prevalence of child diarrhea, which may limit the reliability of the findings in this study. Second, each cluster or primary sampling unit that was used to assess the geographic distribution of high-risk WASH behaviors included a varying number of children, which might not produce a reliable estimate. The prevalence of high-risk WASH practices estimated at the cluster level therefore needs to be assessed by aggregating some of the clusters to be more reliable. By conducting a hot spot analysis in which the mean prevalence of multiple clusters was compared with the mean prevalence of all clusters, this study minimized the risk of producing unreliable findings at the cluster level. Third, the month of data collection as a proxy measure for dry and rainy seasons might not fully control for the effect of seasonality. Fourth, stunting was not included as a potential confounder of the WRI and child diarrhea because it would severely reduce the sample size of this study. Empirical evidence suggests that limited access to WASH and child diarrhea are positively associated with stunting (34, 35). Future studies can estimate the independent effect of the WRI on child diarrhea more accurately by additionally controlling for stunting. Fifth, this study did not collect or examine qualitative data, which could provide contextual information and contribute to explaining why high-risk WASH practices and child diarrhea did not overlap geographically. Using a mixed-method approach may be helpful to obtain a holistic perspective of WASH-related issues (36). Sixth, this study analyzed data from the Uganda DHS 2011, the most recent data set available for the country. Yet, 5 years have passed since the data collection. Future studies with more recent data may guide public health actions more accurately and effectively. Finally, the cross-sectional design of this study cannot establish the temporality of variables or rule out the possibility of reverse causality. Because of severe cases of child diarrhea, households could have changed the source of their drinking water, improved sanitation practice, or created a handwashing place for disease prevention purposes. In this case, the relationship between the dependent and independent variables can be reversed. However, little empirical evidence exists to corroborate this possibility.

## Conclusion

This study revealed the geographic disparities in WASH access and practices that affect Ugandan children under 5 years of age. Large geographic differences in the prevalence of drinking untreated surface water, open defecation, absence of a place for handwashing, and water collection labor were found. In Uganda, WASH interventions should be planned and implemented for the most affected geographic areas based on the findings of this study. More specifically, relevant ministries (e.g. Ministry of Health, Ministry of Water and Environment), civil society organizations, and research institutions may collaborate with one another to implement targeted interventions. This collaboration may also contribute to measuring WASH practices at small geographic units and guiding future public health efforts. The high-risk WASH practices, however, were not found to be associated with child diarrhea, potentially because of recall and reporting biases. Future studies applying experimental designs and different recall periods of child diarrhea may provide useful insights into how high-risk WASH practices are associated with child morbidity and mortality. The potential utility of the WRI can be also examined for different health outcomes than child diarrhea. Although access to adequate WASH conditions needs to be monitored at the national and global level, it is vital to address geographic disparities within each country to target the most vulnerable populations for greater investments.

## Paper context

To the best knowledge of authors, this is the first study that explored geographic distributions of four high-risk WASH practices, including drinking surface water without any treatment, open defecation, lack of handwashing facilities for the household, and water collection time greater than 30 min, at the primary sampling unit level in Uganda. This study contributes to identifying the population bearing the highest WASH-induced burden in small geographic areas for future interventions.

## References

[1] Liu L, Oza S, Hogan D, Perin J, Rudan I, Lawn JE (2015). Global, regional, and national causes of child mortality in 2000–13, with projections to inform post-2015 priorities: an updated systematic analysis. Lancet.

[2] Institute for Health Metrics and Evaluation (2015). GBD compare.

[3] Prüss-Ustün A, Bartram J, Clasen T, Colford JM, Cumming O, Curtis V (2014). Burden of disease from inadequate water, sanitation and hygiene in low- and middle-income settings: a retrospective analysis of data from 145 countries. Trop Med Int Health.

[4] Walker CL, Rudan I, Liu L, Nair H, Theodoratou E, Bhutta ZA (2013). Global burden of childhood pneumonia and diarrhoea. Lancet.

[5] Ashbolt NJ (2004). Microbial contamination of drinking water and disease outcomes in developing regions. Toxicology.

[6] Wagner EG, Lanoix JN (1958). Excreta disposal for rural areas and small communities.

[7] Kotloff KL, Nataro JP, Blackwelder WC, Nasrin D, Farag TH, Panchalingam S (2013). Burden and aetiology of diarrhoeal disease in infants and young children in developing countries (the Global Enteric Multicenter Study, GEMS): a prospective, case-control study. Lancet.

[8] Almedom AS, Blumenthal U, Manderson L (1997). Hygiene evaluation procedures: approaches and methods for assessing water- and sanitation-related hygiene practices.

[9] Fewtrell L, Kaufmann RB, Kay D, Enanoria W, Haller L, Colford JM (2005). Water, sanitation, and hygiene interventions to reduce diarrhoea in less developed countries: a systematic review and meta-analysis. Lancet Infect Dis.

[10] Cairncross S, Hunt C, Boisson S, Bostoen K, Curtis V, Fung IC (2010). Water, sanitation and hygiene for the prevention of diarrhoea. Int J Epidemiol.

[11] UNICEF, WHO (2015). Progress on sanitation and drinking water: 2015 update and MDG assessment.

[12] Pickering AJ, Davis J (2012). Freshwater availability and water fetching distance affect child health in sub-Saharan Africa. Environ Sci Technol.

[13] Howard G, Bartram J (2003). Domestic water quantity, service, level and health.

[14] Freeman MC, Stocks ME, Cumming O, Jeandron A, Higgins JP, Wolf J (2014). Hygiene and health: systematic review of handwashing practices worldwide and update of health effects. Trop Med Int Health.

[15] Pullan RL, Freeman MC, Gething PW, Brooker SJ (2014). Geographical inequalities in use of improved drinking water supply and sanitation across sub-Saharan Africa: mapping and spatial analysis of cross-sectional survey data. PLoS Med.

[16] Cronk R, Luh J, Meier BM, Bartram J (2015). The WASH Performance Index report. http://waterinstitute.unc.edu/wash-performance-index-report/.

[17] Garriga RG, Foguet AP (2013). Unravelling the linkages between water, sanitation, hygiene and rural poverty: the WASH Poverty Index. Water Resour Manag.

[18] Webb AL, Stein AD, Ramakrishnan U, Hertzberg VS, Urizar M, Martorell R (2006). A simple index to measure hygiene behaviours. Int J Epidemiol.

[19] Uganda Bureau of Statistics (UBOS), ICF International (2012). Uganda Demographic and Health Survey 2011.

[20] Corsi DJ, Neuman M, Finlay JE, Subramanian SV (2012). Demographic and Health Surveys: a profile. Int J Epidemiol.

[21] Ram P (2013). Practical guidance for measuring handwashing behavior: update.

[22] Rustein SO, Johnson K (2004). The DHS wealth index: DHS comparative reports No. 6.

[23] Rheingans R, Anderson JD, Luyendijk R, Cumming O (2014). Measuring disparities in sanitation access: does the measure matter?. Trop Med Int Health.

[24] Ord JK, Getis A (1995). Local spatial autocorrelation statistics: distributional issues and an application. Geogr Anal.

[25] Mitchell A (2005). The ESRI guide to GIS analysis, volume 2: spatial measurements and statistics.

[26] ICF International (2011). Demographic and Health Surveys methodology – questionnaires: household, woman's and man's.

[27] Coombes Y, Devine J (2010). Introducing FOAM: a framework to analyze handwashing behaviors to design effective handwashing programs.

[28] Curtis VA, Danquah LO, Aunger RV (2009). Planned, motivated and habitual hygiene behaviour: an eleven country review. Health Educ Res.

[29] Curtis V, Schmidt W, Luby S, Florez R, Touré O, Biran A (2011). Hygiene: new hopes, new horizons. Lancet Infect Dis.

[30] Dreibelbis R, Winch PJ, Leontsini E, Hulland KR, Ram PK, Unicomb L (2013). The Integrated Behavioural Model for Water, Sanitation, and Hygiene: a systematic review of behavioural models and a framework for designing and evaluating behaviour change interventions in infrastructure-restricted settings. BMC Public Health.

[31] Manesh AO, Sheldon TA, Pickett KE, Carr-Hill R (2008). Accuracy of child morbidity data in demographic and health surveys. Int J Epidemiol.

[32] Ssenyonga R, Muwonge R, Twebaze FBN, Mutyabule R (2009). Determinants of acute diarrhoea in children aged 0–5 years in Uganda. East Afr Med J.

[33] Arnold BF, Galiani S, Ram PK, Hubbard AE, Briceño B, Gertler PJ (2013). Optimal recall period for caregiver-reported illness in risk factor and intervention studies: a multicountry study. Am J Epidemiol.

[34] Richard SA, Black RE, Gilman RH, Guerrant RL, Kang G, Lanata CF (2013). Diarrhea in early childhood: short-term association with weight and long-term association with length. Am J Epidemiol.

[35] Ngure FM, Reid BM, Humphrey JH, Mbuya MN, Pelto G, Stoltzfus RJ (2014). Water, sanitation, and hygiene (WASH), environmental enteropathy, nutrition, and early child development: making the links. Ann N Y Acad Sci.

[36] Anthonj C, Rechenburg A, Kistemann T (2016). Water, sanitation and hygiene in wetlands. A case study from the Ewaso Narok Swamp, Kenya. Int J Hyg Environ Health.

